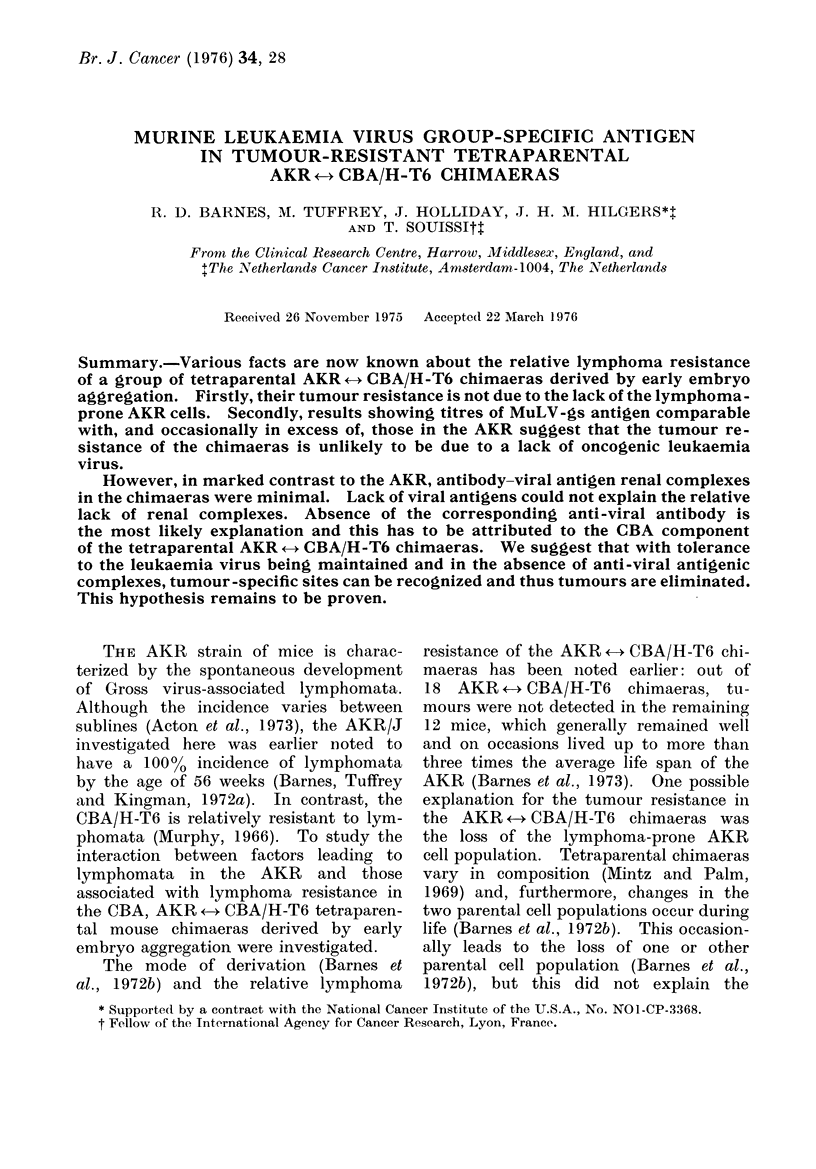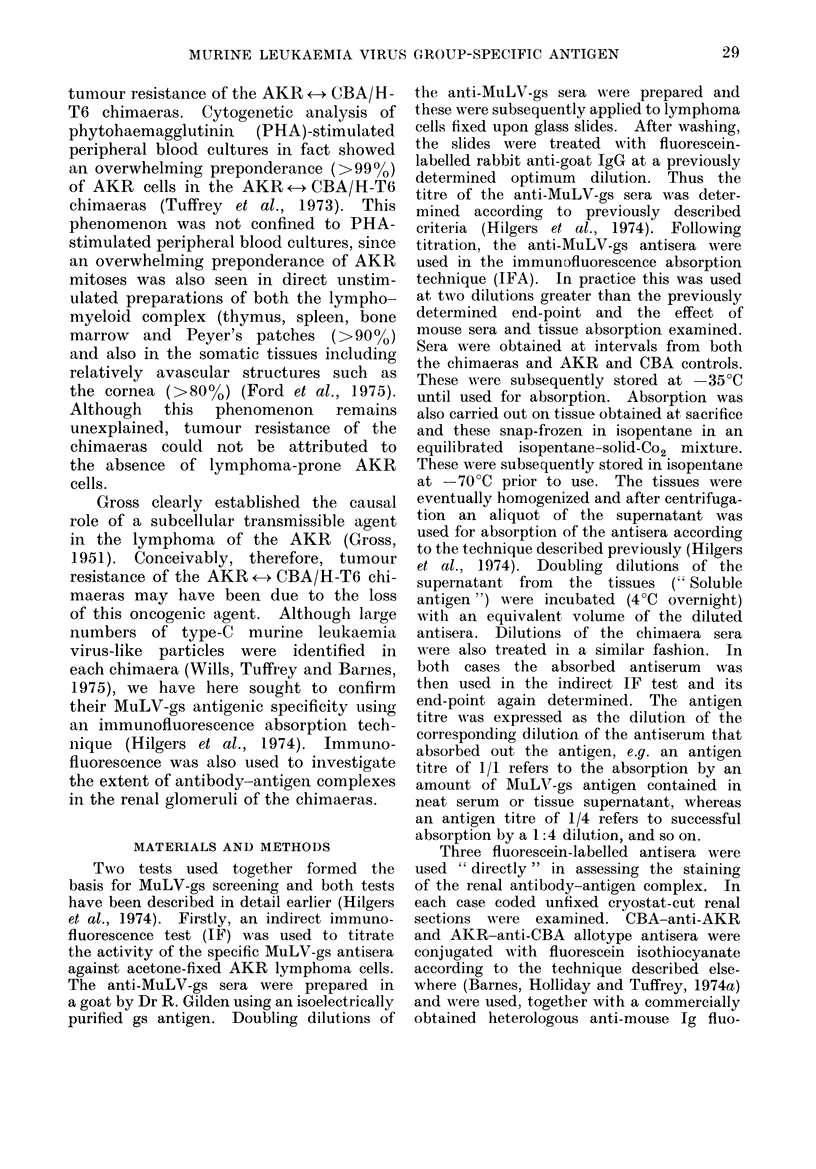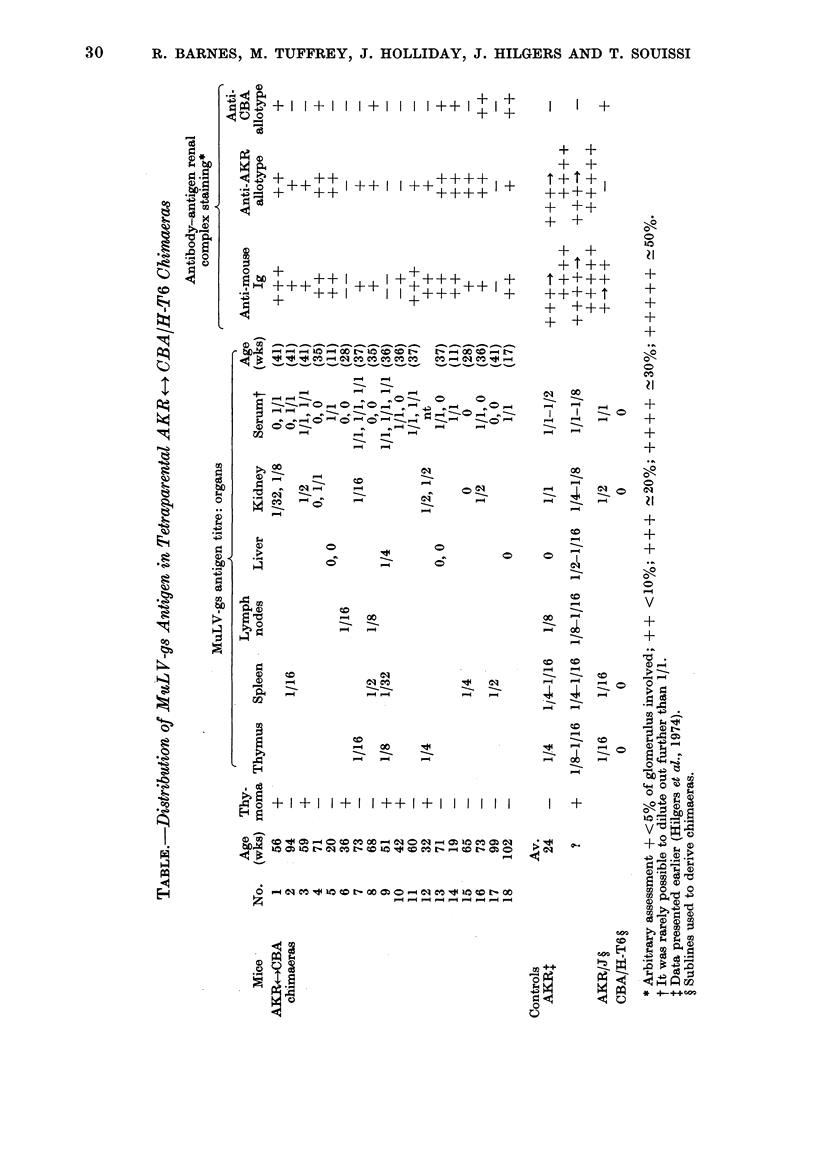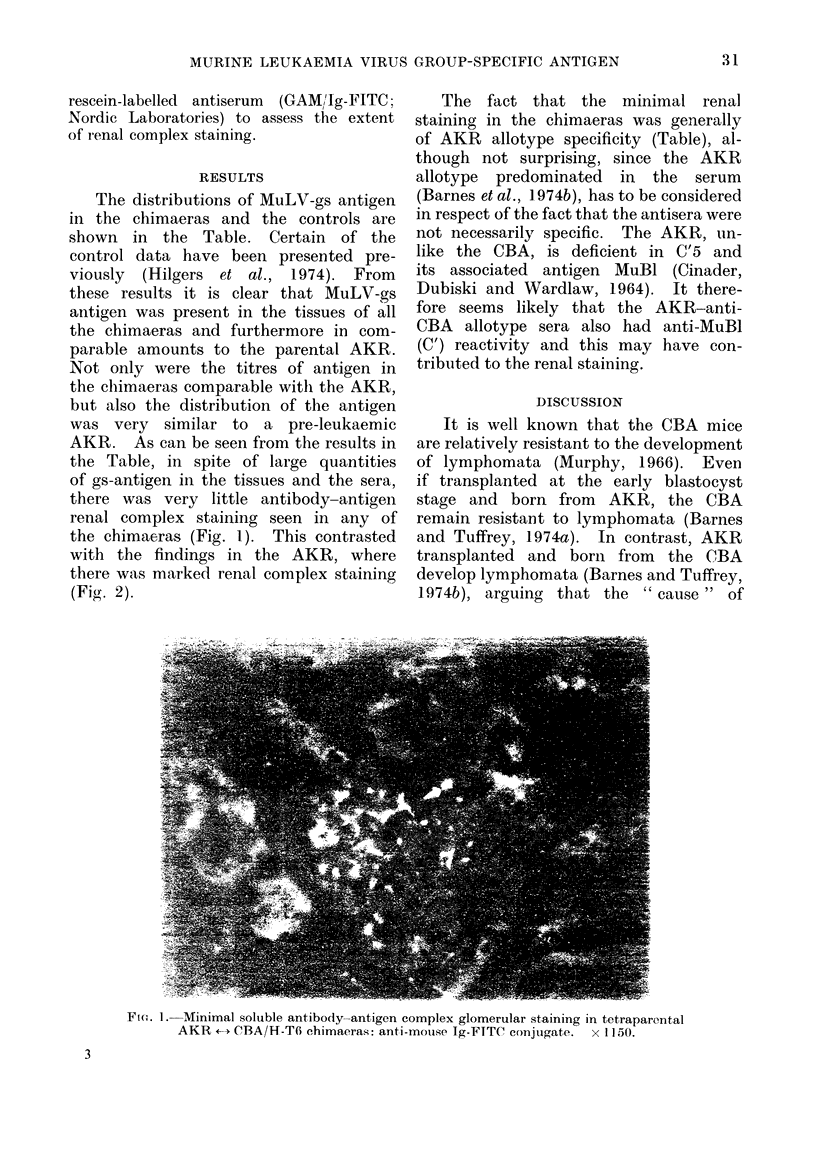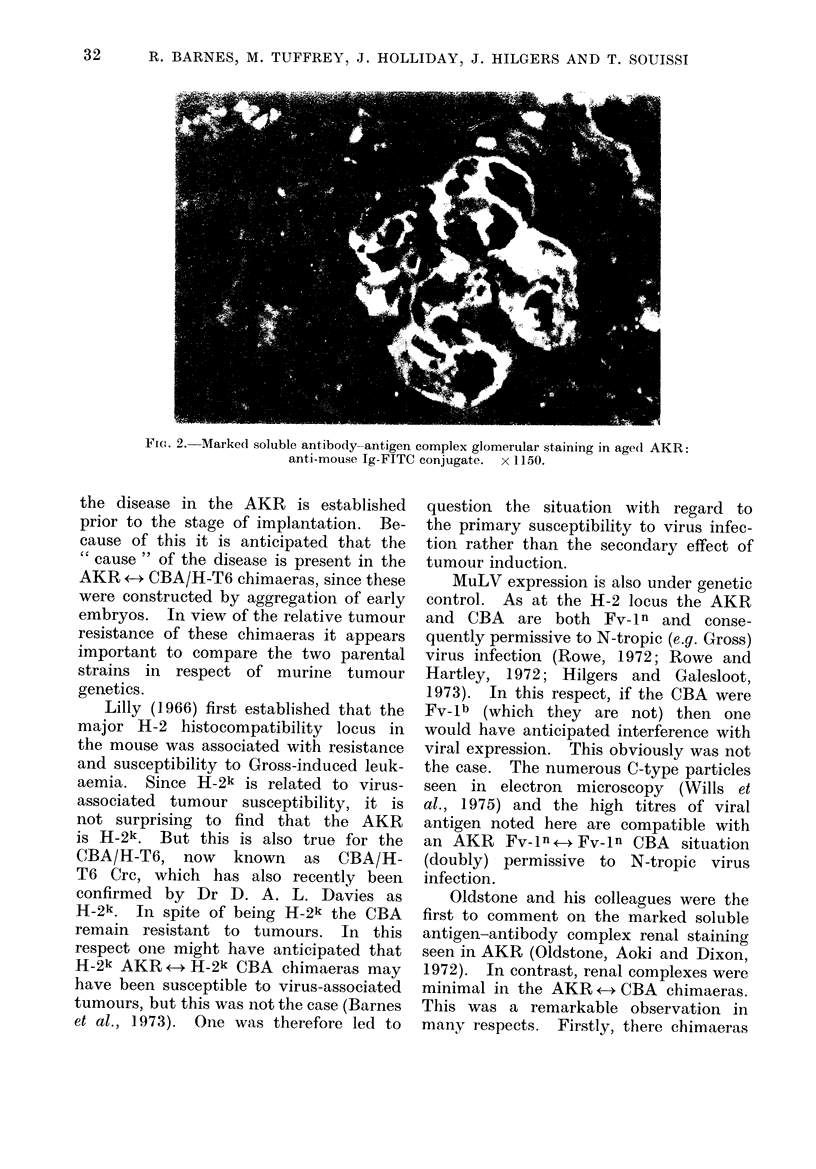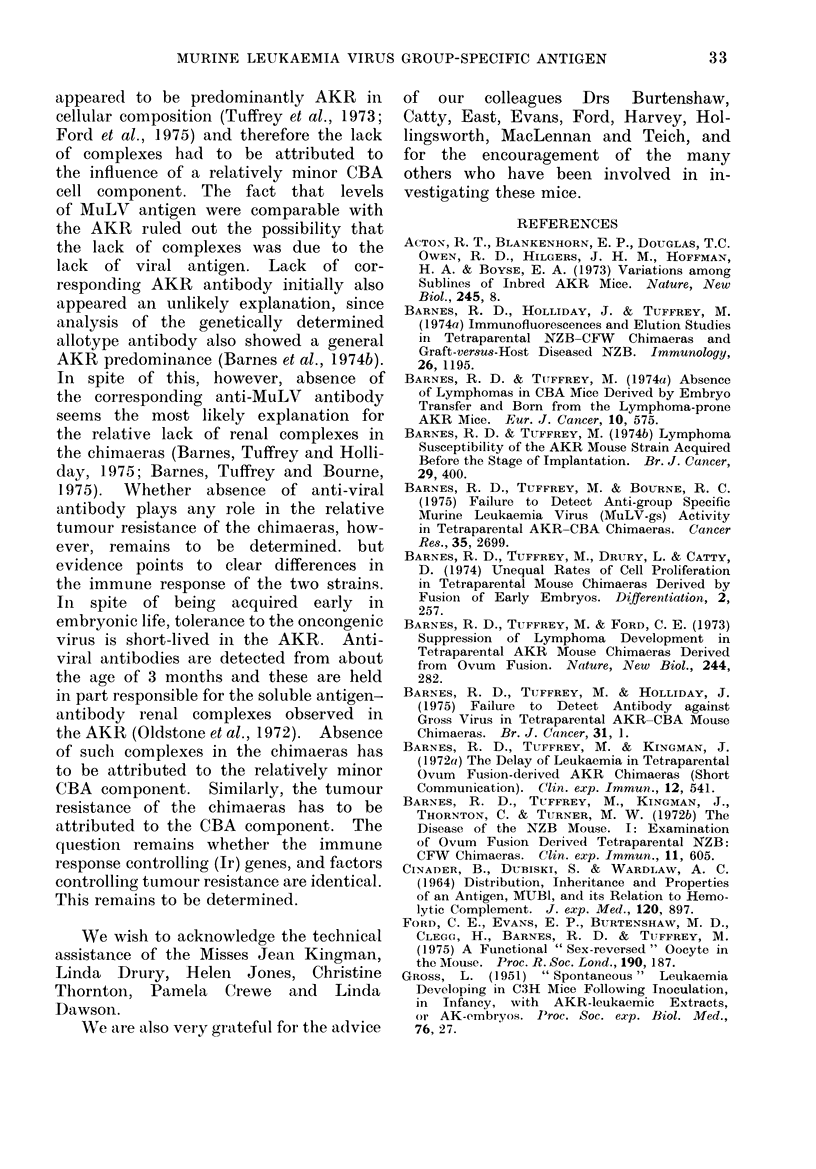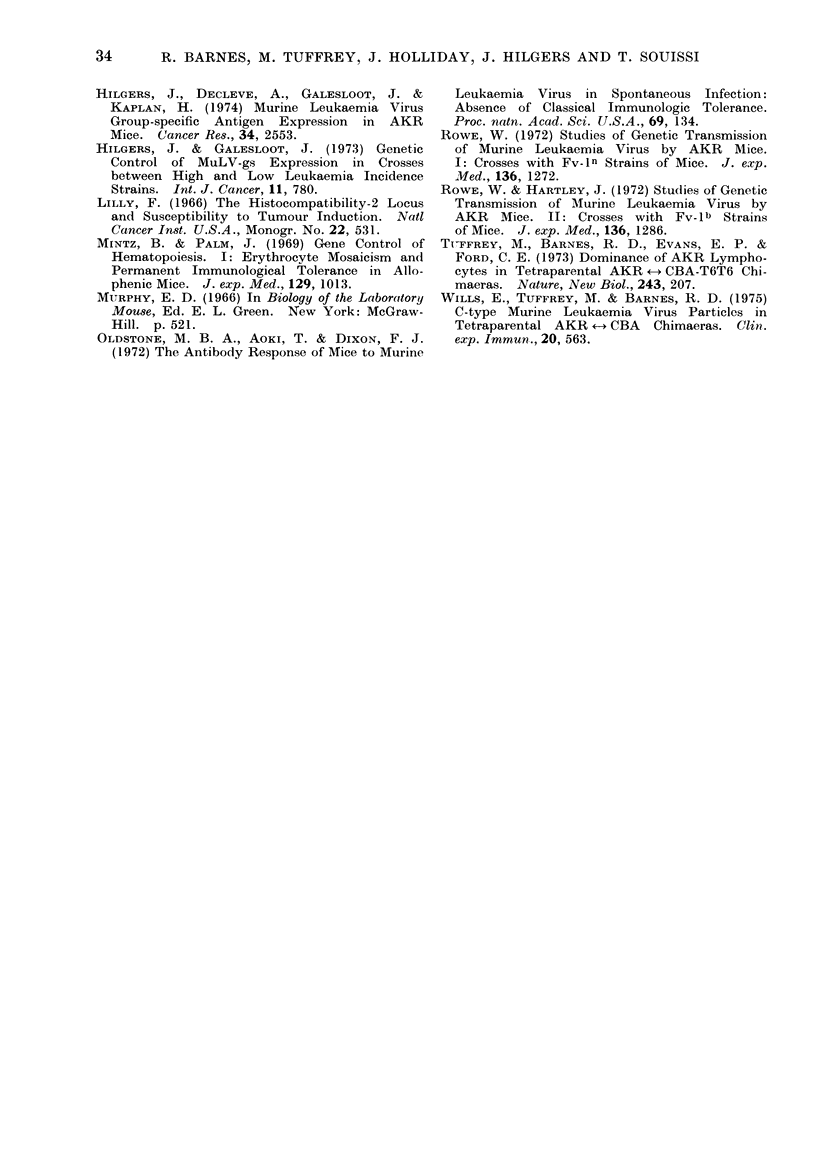# Murine leukaemia virus group-specific antigen in tumor-resistant tetraparental AKR reversible CBA/H-T6 chimaeras.

**DOI:** 10.1038/bjc.1976.117

**Published:** 1976-07

**Authors:** R. D. Barnes, M. Tuffrey, J. Holliday, J. H. Hilgers, T. Souissi

## Abstract

**Images:**


### MURINE LEUKAEMIA VIRUS GROUP-SPECIFIC ANTIGEN IN TUMOUR-RESISTANT TETRAPARENTAL AKR ↔ CBA/H-T6 CHIMAERAS

R. D. BARNES, M. TUFFREY, J. HOLLIDAY, J. H. M. HILGERS*‡ AND T. SOUISSI†‡

*From the Clinical Research Centre, Harrow, Middlesex, England, and ‡The Netherlands Cancer Institute, Amsterdam-1004, The Netherlands*

Received 26 November 1975 Accepted 22 March 1976

**Summary.—Various facts are now known about the relative lymphoma resistance of a group of tetraparental AKR ↔ CBA/H-T6 chimaeras derived by early embryo aggregation. Firstly, their tumour resistance is not due to the lack of the lymphoma-prone AKR cells. Secondly, results showing titres of MuLV-gs antigen comparable with, and occasionally in excess of, those in the AKR suggest that the tumour resistance of the chimaeras is unlikely to be due to a lack of oncogenic leukaemia virus.**

**However, in marked contrast to the AKR, antibody–viral antigen renal complexes in the chimaeras were minimal. Lack of viral antigens could not explain the relative lack of renal complexes. Absence of the corresponding anti-viral antibody is the most likely explanation and this has to be attributed to the CBA component of the tetraparental AKR ↔ CBA/H-T6 chimaeras. We suggest that with tolerance to the leukaemia virus being maintained and in the absence of anti-viral antigenic complexes, tumour-specific sites can be recognized and thus tumours are eliminated. This hypothesis remains to be proven.**

THE AKR strain of mice is characterized by the spontaneous development of Gross virus-associated lymphomata. Although the incidence varies between sublines (Acton *et al.*, 1973), the AKR/J investigated here was earlier noted to have a 100% incidence of lymphomata by the age of 56 weeks (Barnes, Tuffrey and Kingman, 1972*a*). In contrast, the CBA/H-T6 is relatively resistant to lymphomata (Murphy, 1966). To study the interaction between factors leading to lymphomata in the AKR and those associated with lymphoma resistance in the CBA, AKR ↔ CBA/H-T6 tetraparental mouse chimaeras derived by early embryo aggregation were investigated.

The mode of derivation (Barnes *et al.*, 1972*b*) and the relative lymphoma resistance of the AKR ↔ CBA/H-T6 chimaeras has been noted earlier: out of 18 AKR ↔ CBA/H-T6 chimaeras, tumours were not detected in the remaining 12 mice, which generally remained well and on occasions lived up to more than three times the average life span of the AKR (Barnes *et al.*, 1973). One possible explanation for the tumour resistance in the AKR ↔ CBA/H-T6 chimaeras was the loss of the lymphoma-prone AKR cell population. Tetraparental chimaeras vary in composition (Mintz and Palm, 1969) and, furthermore, changes in the two parental cell populations occur during life (Barnes *et al.*, 1972*b*). This occasionally leads to the loss of one or other parental cell population (Barnes *et al.*, 1972*b*), but this did not explain the

* Supported by a contract with the National Cancer Institute of the U.S.A., No. NO1-CP-3368.
† Fellow of the International Agency for Cancer Research, Lyon, France.

tumour resistance of the AKR ↔ CBA/H-T6 chimaeras. Cytogenetic analysis of phytohaemagglutinin (PHA)-stimulated peripheral blood cultures in fact showed an overwhelming preponderance (>99%) of AKR cells in the AKR ↔ CBA/H-T6 chimaeras (Tuffrey *et al.*, 1973). This phenomenon was not confined to PHA-stimulated peripheral blood cultures, since an overwhelming preponderance of AKR mitoses was also seen in direct unstimulated preparations of both the lymphomyeloid complex (thymus, spleen, bone marrow and Peyer's patches (>90%) and also in the somatic tissues including relatively avascular structures such as the cornea (>80%) (Ford *et al.*, 1975). Although this phenomenon remains unexplained, tumour resistance of the chimaeras could not be attributed to the absence of lymphoma-prone AKR cells.

Gross clearly established the causal role of a subcellular transmissible agent in the lymphoma of the AKR (Gross, 1951). Conceivably, therefore, tumour resistance of the AKR ↔ CBA/H-T6 chimaeras may have been due to the loss of this oncogenic agent. Although large numbers of type-C murine leukaemia virus-like particles were identified in each chimaera (Wills, Tuffrey and Barnes, 1975), we have here sought to confirm their MuLV-gs antigenic specificity using an immunofluorescence absorption technique (Hilgers *et al.*, 1974). Immunofluorescence was also used to investigate the extent of antibody–antigen complexes in the renal glomeruli of the chimaeras.

### MATERIALS AND METHODS

Two tests used together formed the basis for MuLV-gs screening and both tests have been described in detail earlier (Hilgers *et al.*, 1974). Firstly, an indirect immunofluorescence test (IF) was used to titrate the activity of the specific MuLV-gs antisera against acetone-fixed AKR lymphoma cells. The anti-MuLV-gs sera were prepared in a goat by Dr R. Gilden using an isoelectrically purified gs antigen. Doubling dilutions of the anti-MuLV-gs sera were prepared and these were subsequently applied to lymphoma cells fixed upon glass slides. After washing, the slides were treated with fluorescein-labelled rabbit anti-goat IgG at a previously determined optimum dilution. Thus the titre of the anti-MuLV-gs sera was determined according to previously described criteria (Hilgers *et al.*, 1974). Following titration, the anti-MuLV-gs antisera were used in the immunofluorescence absorption technique (IFA). In practice this was used at two dilutions greater than the previously determined end-point and the effect of mouse sera and tissue absorption examined. Sera were obtained at intervals from both the chimaeras and AKR and CBA controls. These were subsequently stored at −35°C until used for absorption. Absorption was also carried out on tissue obtained at sacrifice and these snap-frozen in isopentane in an equilibrated isopentane-solid-$Co_2$ mixture. These were subsequently stored in isopentane at −70°C prior to use. The tissues were eventually homogenized and after centrifugation an aliquot of the supernatant was used for absorption of the antisera according to the technique described previously (Hilgers *et al.*, 1974). Doubling dilutions of the supernatant from the tissues (" Soluble antigen ") were incubated (4°C overnight) with an equivalent volume of the diluted antisera. Dilutions of the chimaera sera were also treated in a similar fashion. In both cases the absorbed antiserum was then used in the indirect IF test and its end-point again determined. The antigen titre was expressed as the dilution of the corresponding dilution of the antiserum that absorbed out the antigen, *e.g.* an antigen titre of 1/1 refers to the absorption by an amount of MuLV-gs antigen contained in neat serum or tissue supernatant, whereas an antigen titre of 1/4 refers to successful absorption by a 1:4 dilution, and so on.

Three fluorescein-labelled antisera were used " directly " in assessing the staining of the renal antibody–antigen complex. In each case coded unfixed cryostat-cut renal sections were examined. CBA–anti-AKR and AKR–anti-CBA allotype antisera were conjugated with fluorescein isothiocyanate according to the technique described elsewhere (Barnes, Holliday and Tuffrey, 1974*a*) and were used, together with a commercially obtained heterologous anti-mouse Ig fluo-

TABLE.—*Distribution of MuLV-gs Antigen in Tetraparental AKR ↔ CBA/H-T6 Chimaeras*

| | | | | MuLV-gs antigen titre: organs | | | | | | | Antibody–antigen renal complex staining* | | |
|---|---|---|---|---|---|---|---|---|---|---|---|---|---|
| Mice | No. | Age (wks) | Thy-moma | Thymus | Spleen | Lymph nodes | Liver | Kidney | Serum† | Age (wks) | Anti-mouse Ig | Anti-AKR allotype | Anti-CBA allotype |
| AKR↔CBA chimaeras | 1 | 56 | + | | | | | 1/32, 1/8 | 0, 1/1 | (41) | +++ | ++ | + |
| | 2 | 94 | − | | 1/16 | | | | 0, 1/1 | (41) | + | + | − |
| | 3 | 59 | + | | | | | 1/2 | 1/1, 1/1 | (41) | + | + | − |
| | 4 | 71 | − | | | | | 0, 1/1 | 0, 0 | (35) | ++ | ++ | + |
| | 5 | 20 | − | | | | 0, 0 | | 1/1 | (11) | ++ | ++ | − |
| | 6 | 36 | + | | | 1/16 | | | 0, 0 | (28) | −− | − | − |
| | 7 | 73 | − | 1/16 | | | | 1/16 | 1/1, 1/1, 1/1 | (37) | + | + | − |
| | 8 | 68 | − | | 1/2 | 1/8 | | | 0, 0 | (35) | + | + | + |
| | 9 | 51 | + | 1/8 | 1/32 | | 1/4 | | 1/1, 1/1, 1/1 | (36) | −− | − | − |
| | 10 | 42 | + | | | | | | 1/1, 0 | (36) | −+ | − | − |
| | 11 | 60 | − | | | | | | 1/1, 1/1 | (37) | +++ | + | − |
| | 12 | 32 | + | 1/4 | | | | 1/2, 1/2 | nt | | ++ | + | − |
| | 13 | 71 | − | | | | 0, 0 | | 1/1, 0 | (37) | ++ | ++ | + |
| | 14 | 19 | − | | | | | | 1/1 | (11) | ++ | ++ | + |
| | 15 | 65 | − | | 1/4 | | | 0 | 0 | (28) | + | ++ | − |
| | 16 | 73 | − | | | | | 1/2 | 1/1, 0 | (36) | + | ++ | ++ |
| | 17 | 99 | − | | 1/2 | | | | 0, 0 | (41) | − | − | − |
| | 18 | 102 | − | | | | 0 | | 1/1 | (17) | ++ | + | ++ |
| Controls AKR‡ | | Av. 24 | − | 1/4 | 1/4–1/16 | 1/8 | 0 | 1/1 | 1/1–1/2 | | +++→ ++++ | +++→ ++++ | − |
| | | ? | + | 1/8–1/16 | 1/4–1/16 | 1/8–1/16 | 1/2–1/16 | 1/4–1/8 | 1/1–1/8 | | ++++→ +++++ | +++→ +++++ | − |
| AKR/J§ | | | | 1/16 | 1/16 | | | 1/2 | 1/1 | | +→++ | − | + |
| CBA/H-T6§ | | | | 0 | 0 | | | 0 | 0 | | | | |

* Arbitrary assessment + <5% of glomerulus involved; ++ <10%; +++ ≃20%; ++++ ≃30%; +++++ ≃50%.

† It was rarely possible to dilute out further than 1/1.

‡ Data presented earlier (Hilgers *et al.*, 1974).

§ Sublines used to derive chimaeras.

rescein-labelled antiserum (GAM/Ig-FITC; Nordic Laboratories) to assess the extent of renal complex staining.

### RESULTS

The distributions of MuLV-gs antigen in the chimaeras and the controls are shown in the Table. Certain of the control data have been presented previously (Hilgers *et al.*, 1974). From these results it is clear that MuLV-gs antigen was present in the tissues of all the chimaeras and furthermore in comparable amounts to the parental AKR. Not only were the titres of antigen in the chimaeras comparable with the AKR, but also the distribution of the antigen was very similar to a pre-leukaemic AKR. As can be seen from the results in the Table, in spite of large quantities of gs-antigen in the tissues and the sera, there was very little antibody–antigen renal complex staining seen in any of the chimaeras (Fig. 1). This contrasted with the findings in the AKR, where there was marked renal complex staining (Fig. 2).

The fact that the minimal renal staining in the chimaeras was generally of AKR allotype specificity (Table), although not surprising, since the AKR allotype predominated in the serum (Barnes *et al.*, 1974*b*), has to be considered in respect of the fact that the antisera were not necessarily specific. The AKR, unlike the CBA, is deficient in C′5 and its associated antigen MuBl (Cinader, Dubiski and Wardlaw, 1964). It therefore seems likely that the AKR–anti-CBA allotype sera also had anti-MuBl (C′) reactivity and this may have contributed to the renal staining.

### DISCUSSION

It is well known that the CBA mice are relatively resistant to the development of lymphomata (Murphy, 1966). Even if transplanted at the early blastocyst stage and born from AKR, the CBA remain resistant to lymphomata (Barnes and Tuffrey, 1974*a*). In contrast, AKR transplanted and born from the CBA develop lymphomata (Barnes and Tuffrey, 1974*b*), arguing that the “ cause ” of

FIG. 1.—Minimal soluble antibody–antigen complex glomerular staining in tetraparental AKR ↔ CBA/H-T6 chimaeras: anti-mouse Ig-FITC conjugate. × 1150.

FIG. 2.—Marked soluble antibody–antigen complex glomerular staining in aged AKR: anti-mouse Ig-FITC conjugate. × 1150.

the disease in the AKR is established prior to the stage of implantation. Because of this it is anticipated that the " cause " of the disease is present in the AKR ↔ CBA/H-T6 chimaeras, since these were constructed by aggregation of early embryos. In view of the relative tumour resistance of these chimaeras it appears important to compare the two parental strains in respect of murine tumour genetics.

Lilly (1966) first established that the major H-2 histocompatibility locus in the mouse was associated with resistance and susceptibility to Gross-induced leukaemia. Since $H\text{-}2^k$ is related to virus-associated tumour susceptibility, it is not surprising to find that the AKR is $H\text{-}2^k$. But this is also true for the CBA/H-T6, now known as CBA/H-T6 Crc, which has also recently been confirmed by Dr D. A. L. Davies as $H\text{-}2^k$. In spite of being $H\text{-}2^k$ the CBA remain resistant to tumours. In this respect one might have anticipated that $H\text{-}2^k$ AKR ↔ $H\text{-}2^k$ CBA chimaeras may have been susceptible to virus-associated tumours, but this was not the case (Barnes *et al.*, 1973). One was therefore led to question the situation with regard to the primary susceptibility to virus infection rather than the secondary effect of tumour induction.

MuLV expression is also under genetic control. As at the H-2 locus the AKR and CBA are both $Fv\text{-}1^n$ and consequently permissive to N-tropic (*e.g.* Gross) virus infection (Rowe, 1972; Rowe and Hartley, 1972; Hilgers and Galesloot, 1973). In this respect, if the CBA were $Fv\text{-}1^b$ (which they are not) then one would have anticipated interference with viral expression. This obviously was not the case. The numerous C-type particles seen in electron microscopy (Wills *et al.*, 1975) and the high titres of viral antigen noted here are compatible with an AKR $Fv\text{-}1^n$ ↔ $Fv\text{-}1^n$ CBA situation (doubly) permissive to N-tropic virus infection.

Oldstone and his colleagues were the first to comment on the marked soluble antigen–antibody complex renal staining seen in AKR (Oldstone, Aoki and Dixon, 1972). In contrast, renal complexes were minimal in the AKR ↔ CBA chimaeras. This was a remarkable observation in many respects. Firstly, there chimaeras appeared to be predominantly AKR in cellular composition (Tuffrey *et al.*, 1973; Ford *et al.*, 1975) and therefore the lack of complexes had to be attributed to the influence of a relatively minor CBA cell component. The fact that levels of MuLV antigen were comparable with the AKR ruled out the possibility that the lack of complexes was due to the lack of viral antigen. Lack of corresponding AKR antibody initially also appeared an unlikely explanation, since analysis of the genetically determined allotype antibody also showed a general AKR predominance (Barnes *et al.*, 1974*b*). In spite of this, however, absence of the corresponding anti-MuLV antibody seems the most likely explanation for the relative lack of renal complexes in the chimaeras (Barnes, Tuffrey and Holliday, 1975; Barnes, Tuffrey and Bourne, 1975). Whether absence of anti-viral antibody plays any role in the relative tumour resistance of the chimaeras, however, remains to be determined. but evidence points to clear differences in the immune response of the two strains. In spite of being acquired early in embryonic life, tolerance to the oncongenic virus is short-lived in the AKR. Anti-viral antibodies are detected from about the age of 3 months and these are held in part responsible for the soluble antigen–antibody renal complexes observed in the AKR (Oldstone *et al.*, 1972). Absence of such complexes in the chimaeras has to be attributed to the relatively minor CBA component. Similarly, the tumour resistance of the chimaeras has to be attributed to the CBA component. The question remains whether the immune response controlling (Ir) genes, and factors controlling tumour resistance are identical. This remains to be determined.

We wish to acknowledge the technical assistance of the Misses Jean Kingman, Linda Drury, Helen Jones, Christine Thornton, Pamela Crewe and Linda Dawson.

We are also very grateful for the advice of our colleagues Drs Burtenshaw, Catty, East, Evans, Ford, Harvey, Hollingsworth, MacLennan and Teich, and for the encouragement of the many others who have been involved in investigating these mice.

### REFERENCES

Acton, R. T., Blankenhorn, E. P., Douglas, T.C. Owen, R. D., Hilgers, J. H. M., Hoffman, H. A. & Boyse, E. A. (1973) Variations among Sublines of Inbred AKR Mice. *Nature, New Biol.*, **245**, 8.

Barnes, R. D., Holliday, J. & Tuffrey, M. (1974*a*) Immunofluorescences and Elution Studies in Tetraparental NZB–CFW Chimaeras and Graft-*versus*-Host Diseased NZB. *Immunology*, **26**, 1195.

Barnes, R. D. & Tuffrey, M. (1974*a*) Absence of Lymphomas in CBA Mice Derived by Embryo Transfer and Born from the Lymphoma-prone AKR Mice. *Eur. J. Cancer*, **10**, 575.

Barnes, R. D. & Tuffrey, M. (1974*b*) Lymphoma Susceptibility of the AKR Mouse Strain Acquired Before the Stage of Implantation. *Br. J. Cancer*, **29**, 400.

Barnes, R. D., Tuffrey, M. & Bourne, R. C. (1975) Failure to Detect Anti-group Specific Murine Leukaemia Virus (MuLV-gs) Activity in Tetraparental AKR–CBA Chimaeras. *Cancer Res.*, **35**, 2699.

Barnes, R. D., Tuffrey, M., Drury, L. & Catty, D. (1974) Unequal Rates of Cell Proliferation in Tetraparental Mouse Chimaeras Derived by Fusion of Early Embryos. *Differentiation*, **2**, 257.

Barnes, R. D., Tuffrey, M. & Ford, C. E. (1973) Suppression of Lymphoma Development in Tetraparental AKR Mouse Chimaeras Derived from Ovum Fusion. *Nature, New Biol.*, **244**, 282.

Barnes, R. D., Tuffrey, M. & Holliday, J. (1975) Failure to Detect Antibody against Gross Virus in Tetraparental AKR–CBA Mouse Chimaeras. *Br. J. Cancer*, **31**, 1.

Barnes, R. D., Tuffrey, M. & Kingman, J. (1972*a*) The Delay of Leukaemia in Tetraparental Ovum Fusion-derived AKR Chimaeras (Short Communication). *Clin. exp. Immun.*, **12**, 541.

Barnes, R. D., Tuffrey, M., Kingman, J., Thornton, C. & Turner, M. W. (1972*b*) The Disease of the NZB Mouse. I: Examination of Ovum Fusion Derived Tetraparental NZB: CFW Chimaeras. *Clin. exp. Immun.*, **11**, 605.

Cinader, B., Dubiski, S. & Wardlaw, A. C. (1964) Distribution, Inheritance and Properties of an Antigen, MUBl, and its Relation to Hemolytic Complement. *J. exp. Med.*, **120**, 897.

Ford, C. E., Evans, E. P., Burtenshaw, M. D., Clegg, H., Barnes, R. D. & Tuffrey, M. (1975) A Functional " Sex-reversed " Oocyte in the Mouse. *Proc. R. Soc. Lond.*, **190**, 187.

Gross, L. (1951) " Spontaneous " Leukaemia Developing in C3H Mice Following Inoculation, in Infancy, with AKR-leukaemic Extracts, or AK-embryos. *Proc. Soc. exp. Biol. Med.*, **76**, 27.

Hilgers, J., Decleve, A., Galesloot, J. & Kaplan, H. (1974) Murine Leukaemia Virus Group-specific Antigen Expression in AKR Mice. *Cancer Res.*, **34**, 2553.

Hilgers, J. & Galesloot, J. (1973) Genetic Control of MuLV-gs Expression in Crosses between High and Low Leukaemia Incidence Strains. *Int. J. Cancer*, **11**, 780.

Lilly, F. (1966) The Histocompatibility-2 Locus and Susceptibility to Tumour Induction. *Natl Cancer Inst. U.S.A.*, Monogr. No. **22**, 531.

Mintz, B. & Palm, J. (1969) Gene Control of Hematopoiesis. I: Erythrocyte Mosaicism and Permanent Immunological Tolerance in Allophenic Mice. *J. exp. Med.*, **129**, 1013.

Murphy, E. D. (1966) In *Biology of the Laboratory Mouse*, Ed. E. L. Green. New York: McGraw-Hill. p. 521.

Oldstone, M. B. A., Aoki, T. & Dixon, F. J. (1972) The Antibody Response of Mice to Murine Leukaemia Virus in Spontaneous Infection: Absence of Classical Immunologic Tolerance. *Proc. natn. Acad. Sci. U.S.A.*, **69**, 134.

Rowe, W. (1972) Studies of Genetic Transmission of Murine Leukaemia Virus by AKR Mice. I: Crosses with Fv-$1^n$ Strains of Mice. *J. exp. Med.*, **136**, 1272.

Rowe, W. & Hartley, J. (1972) Studies of Genetic Transmission of Murine Leukaemia Virus by AKR Mice. II: Crosses with Fv-$1^b$ Strains of Mice. *J. exp. Med.*, **136**, 1286.

Tuffrey, M., Barnes, R. D., Evans, E. P. & Ford, C. E. (1973) Dominance of AKR Lymphocytes in Tetraparental AKR ↔ CBA-T6T6 Chimaeras. *Nature, New Biol.*, **243**, 207.

Wills, E., Tuffrey, M. & Barnes, R. D. (1975) C-type Murine Leukaemia Virus Particles in Tetraparental AKR ↔ CBA Chimaeras. *Clin. exp. Immun.*, **20**, 563.